# Bovine Astrovirus Surveillance in Uruguay Reveals High Detection Rate of a Novel *Mamastrovirus* Species

**DOI:** 10.3390/v12010032

**Published:** 2019-12-27

**Authors:** Matías Castells, Estefany Bertoni, Rubén Darío Caffarena, María Laura Casaux, Carlos Schild, Matías Victoria, Franklin Riet-Correa, Federico Giannitti, Viviana Parreño, Rodney Colina

**Affiliations:** 1 Laboratorio de Virología Molecular, CENUR Litoral Norte, Centro Universitario de Salto, Universidad de la República, Rivera 1350, Salto, Uruguay; 2 Instituto Nacional de Investigación Agropecuaria (INIA), Plataforma de Investigación en Salud Animal, Ruta 50 km 11, La Estanzuela 64988, Colonia, Uruguay; 3 Departamento de Patología y Clínica de Rumiantes y Suinos, Facultad de Veterinaria, Universidad de la República. Alberto Lasplaces 1620, Montevideo, Uruguay; 4 Sección de Virus Gastroentéricos, Instituto de Virología, CICV y A, INTA Castelar, Nicolás Repetto S/N, Buenos Aires 1686, Argentina

**Keywords:** bovine astrovirus, dairy cattle, genetic diversity, prevalence, Uruguay, Mamastrovirus species

## Abstract

Viral infections affecting cattle lead to economic losses to the livestock industry worldwide, but little is known about the circulation, pathogenicity and genetic diversity of enteric bovine astrovirus (BoAstV) in America. The aim of this work was to describe the prevalence and genetic diversity of enteric BoAstV in dairy cattle in Uruguay. A total of 457 fecal and 43 intestinal contents from dairy calves were collected between July 2015 and May 2017 and tested by RT-PCR, followed by sequencing and phylogenetic analyses of the polymerase and capsid regions. Twenty-six percent (128/500) of the samples were positive. Three different species within the *Mamastrovirus* genus were identified, including *Mamastrovirus 28*, *Mamastrovirus 33* (3 samples each) and an unclassified *Mamastrovirus* species (19 samples). The unclassified species was characterized as a novel *Mamastrovirus* species. BoAstV circulates in Uruguayan dairy cattle with a high genetic diversity. The eventual clinicopathological significance of enteric BoAstV infection in cattle needs further investigation.

## 1. Introduction

Astroviruses (AstVs) are small, non-enveloped viruses, of 28 to 30 nm in diameter, with distinctive five- or six-pointed, star-like virions. The genome is composed of positive-sense, single-stranded RNA of 6.3–7.9 kb that includes three open reading frames (ORFs). AstVs are members of the *Astroviridae* family, which is further divided in two genera: *Mamastrovirus* (MAstV) and *Avastrovirus*, infecting mammals and birds, respectively. Bovine astroviruses (BoAstVs) are members of the MAstV genus, detected for the first time in 1978, in calves with enteritis [1]. Initially, BoAstVs did not capture much attention in the scientific community, due to their inability to cause diarrhea in experimentally infected gnotobiotic calves [1], although it was proven that they are indeed capable of infecting the dome epithelial cells of the ileum [2]. In coinfections with other viruses, such as rotavirus and torovirus, the infectious capacity of BoAstV seems to be increased [2], although whether this is clinically relevant, or the virus plays a synergistic role in the development of diarrhea in cattle, needs further clarification. In 2013, the association of a genetically different lineage of BoAstV with neurologic disease and encephalomyelitis in cattle [3], which was recently described in Uruguay [4], resulted in increased interest in studying this group of viruses, along with their molecular epidemiology and pathogenicity. Recently, BoAstV was detected in 4/50 calves with bovine respiratory disease (BRD) and was absent in 50 asymptomatic calves in a case control study, but this viral infection was not statistically associated with BRD [5]. The role of AstV in respiratory disease remains unclear, but there is increasing evidence of detection of AstV in respiratory samples from humans and animals with acute respiratory disease [5,6,7,8].

MAstV is divided into viral species based on genetic differences higher than approximately 0.30 to 0.35 at the protein level in the complete capsid sequence [9]. The current classification of the MAstV genus according to the International Committee on Taxonomy of Viruses (ICTV), includes 19 species (namely MAstV-1 through 19) (https://talk.ictvonline.org/taxonomy/, last access 25th October 2019), although there are up to 33 tentative species, not yet confirmed by the ICTV [9,10]. BoAstVs have not yet been assigned to species. Nevertheless, it has been proposed that they belong to at least six different species: MAstV-13, MAstV-24, MAstV-28, MAstV-29, MAstV-30 and MAstV-33, that are closely related to other AstVs from various host species, indicating possible interspecies transmission events [10,11]. Two serotypes have been recognized [12], but current phylogenetic evidence indicates that the diversity of BoAstVs circulating in cattle is probably greater [10]. The aim of this study was to determine the prevalence of enteric infection by BoAstV and its genetic diversity in Uruguayan dairy cattle.

## 2. Materials and Methods

### 2.1. Samples, RNA Extraction and Reverse Transcription

A total of 500 samples (457 fecal and 43 intestinal contents) obtained from calves belonging to dairy herds of the Uruguayan dairy basin, were collected between July 2015 and May 2017. Samples were diluted 1:10 (*v*:*v*) in phosphate-buffered saline solution, centrifuged at 3000× *g* for 20 min at 4 °C, and supernatants were collected and stored at −80 °C. Viral RNA was extracted from each sample using QIAamp^®^ cador^®^ Pathogen Mini Kit (Qiagen^®^, Hilden, Germany), following the manufacturer’s instructions. Reverse transcription (RT) was carried out with RevertAid^®^ Reverse Transcriptase (Thermo Fischer Scientific^®^, Waltham, MA, USA) and random hexamers primers (Qiagen^®^). All RNAs and cDNAs were stored at −20 °C.

### 2.2. Polymerase Chain Reaction for BoAstV Detection and Sequencing

Polymerase chain reactions (PCR) were performed using MangoMix™ (Bioline^®^, London, UK) and primers that amplify a 432-nucleotide fragment of the polymerase gene of AstV. Briefly, 12.5 μL of MangoMix™, 5 μL of cDNA, 4.5 μL of nuclease-free water, 1 μL of dimethyl sulfoxide, 1 μL of 10 μM BoAstV-F primer and 1 μL of 10 μM BoAstV-R primer were mixed in 0.2 mL PCR tubes. PCR primers sequences and cycling conditions were the same as those described by Tse et al. [13]. PCR products were visualized in 2% agarose gels, 25 (20%). PCR-positive products were purified using PureLink™ Quick Gel Extraction and PCR Purification Combo Kit (Invitrogen^®^, Carlsbad, CA, USA), according to the manufacturer’s instructions, and both DNA strands were sequenced by Macrogen Inc. (Seoul, South Korea). Sequences were deposited in GenBank with accession numbers MH123895-MH123814 and MH123817-MH123921.

### 2.3. Capsid PCR and Sequencing

Complete capsid amplification of strains LVMS681 and LVMS2704 was performed. Primers were designed in order to amplify complete capsid (Table 1), using sequences obtained from GenBank. Briefly, 12.5 μL of MangoMix™, 5 μL of cDNA, 4.5 μL of nuclease-free water, 1 μL of dimethyl sulfoxide, 1 μL of 10 μM primer forward and 1 μL of 10 μM primer reverse were mixed in 0.2 mL PCR tubes and subjected to an initial step of 5 min at 95 °C, followed by 40 cycles of 94 °C for 1 min, 48 °C for 1 min, and 72 °C for 1.5 min, ending with 10 min at 72 °C for final extension. PCR products were visualized in 1–2% agarose gels, purified using PureLink™ Quick Gel Extraction and PCR Purification Combo Kit (Invitrogen^®^), according to the manufacturer’s instructions, and both DNA strands were sequenced by Macrogen Inc. (Seoul, South Korea). Sequences were deposited in GenBank with accession numbers MN200262 and MN200263. In addition, six partial capsid sequences were obtained and deposited in GenBank with accession numbers MN231236-MN231241.

### 2.4. Phylogenetic Analyses

Available sequences of a partial polymerase genomic region of BoAstV and related AstVs from other hosts (344 nucleotides from position 3289 to 3620, reference strain accession number: NC_023631) were downloaded from the GenBank database (Table S1).

For MAstV classification, complete capsid sequences were downloaded from GenBank database (Table S2). The datasets of nucleotide and amino acid sequences were aligned using Clustal W implemented in MEGA 7 software [14]. The substitution model that best fit each alignment and maximum likelihood trees (with the substitution model selected previously) were obtained with W-IQ-TREE (available at http://iqtree.cibiv.univie.ac.at) [15].

### 2.5. Estimates of the Evolutionary Divergence between Sequences

Amino acid p-distances were estimated with MEGA 7 software [14]. Pairwise distances were estimated with the default parameters of MEGA version 7.0.21, and the alignment of complete capsid amino acid sequences, obtained previously for the phylogenetic analysis, was used as input.

## 3. Results

**Figure 1:**
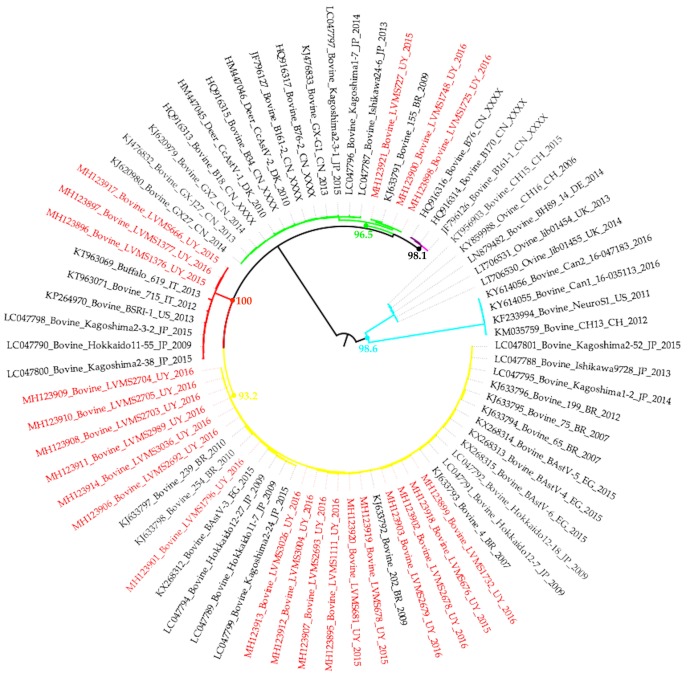
Maximum-likelihood tree constructed with the nucleotide sequences of the partial polymerase genomic region used for BoAstV screening. A 432-nucleotide fragment of the polymerase gene was used for the analysis. TIM2e (AC = AT, CG = GT and equal base frequencies) plus R (FreeRate model, that generalizes the plus Gamma model by relaxing the assumption of Gamma-distributed rates) was used as the nucleotide substitution model that best fitted the data. Branch colors indicate the assigned MAstV species; MAstV-13 (light blue), MAstV-28 (red), MAstV-29 (pink), MAstV-30 (violet), MAstV-33 (green) and MAstV-Unclassified (yellow). Names in red font correspond to Uruguayan strains. Bootstrap values for key nodes are indicated.

BoAstV was detected in 26% (128/500) of the samples screened by PCR targeting the polymerase genomic region. The same frequency of detection was observed in feces (26%, 117/457) and intestinal contents (26%, 11/43). Phylogenetic analysis with the partial polymerase genomic region showed that the Uruguayan strains clustered in three groups: 3/25 (12%) with MAstV-28, and 3/25 (12%) with MAstV-33, while 19/25 (76%) strains formed a monophyletic group with other unclassified strains from all over the world (Figure 1).

**Figure 2:**
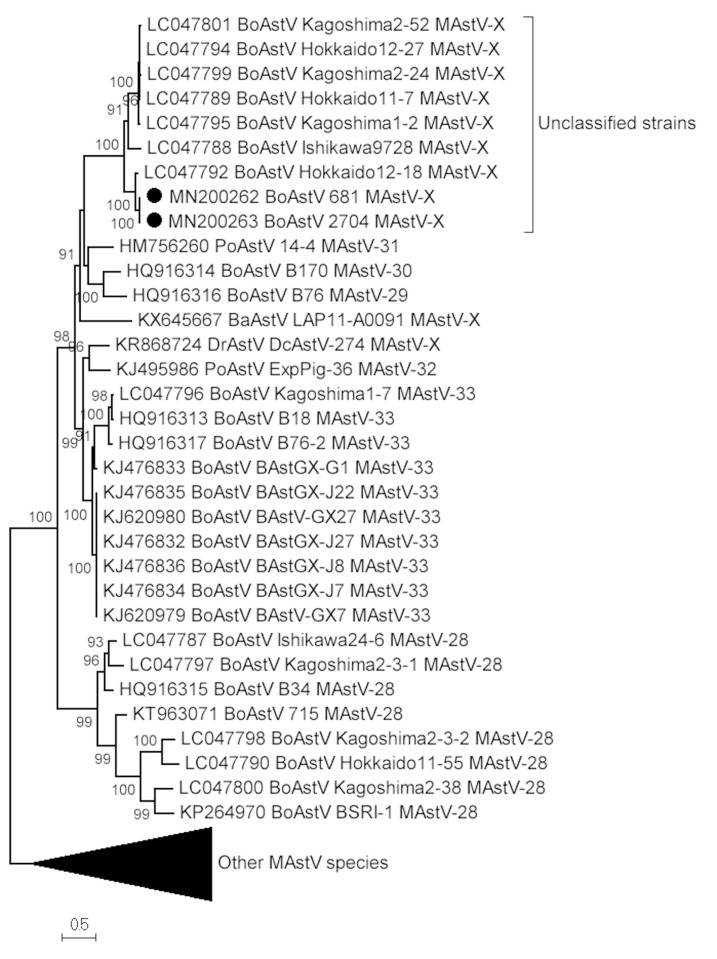
Maximum-likelihood tree constructed with complete capsid amino acid sequences. LG (General matrix) + gamma (with four categories of rates to approximate the gamma distribution) + invariant sites (allows a proportion of invariable sites) was used as the amino acid substitution model that best fit the data. All the assigned and tentatively assigned species of MAstV were included in the analysis (see Table S2); some were condensed for better visualization. The two Uruguayan strains are indicated with black-filled circles. The clade with the unclassified strains is shown. aLRT values higher than 90 are shown.

The phylogenetic analyses with the complete capsid sequences confirmed the clustering of the Uruguayan strains LVMS681 and LVMS2704 in a group with other unclassified strains, both at amino acid and nucleotide levels (Figure 2 and Figure S1). These results were confirmed by the analysis of the p-distance in the complete capsid sequence at the protein level (Table 2). Based on these analyses, LVMS681 and LVMS2704 should be assigned to a new MAstV species by the ICTV.

**Figure 3:**
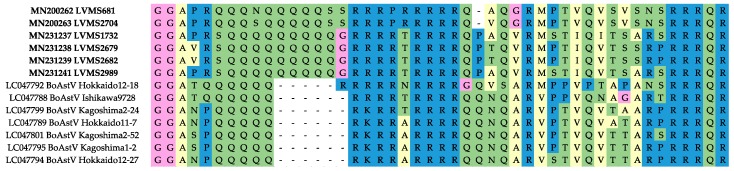
Amino acid alignment of unclassified strains. The amino acid alignment from position 44 to 89 of the capsid protein (LVMS2704 used as reference) is shown.

Based on partial capsid sequences, six additional Uruguayan strains were classified; four within the unclassified MAstV species, one within MAstV-28 and one within MAstV-29 (Figure S2). Uruguayan strains of the unclassified MAstV species showed an insertion of six amino acids (positions 54–59 of the LVMS2704 complete capsid amino acid sequence), when compared with Japanese strains of this species available in the database (Figure 3). This region is characterized by a repetitive sequence of polar uncharged amino acids (mainly glutamine) followed by a repetitive sequence of basic amino acids (mainly arginine); the insertion is in the polar uncharged region and is mainly formed of glutamines (Figure 3). In addition, a deletion of one amino acid was observed in LVMS681 and LVMS2704 (between positions 69 and 70 of the LVMS2704 complete capsid amino acid sequence) (Figure 3).

## 4. Discussion

Enteric infection with animal and human AstVs have been associated with diarrhea [16], however the role of BoAstV as a causative agent of diarrhea in cattle is controversial and has not been extensively studied. Although BoAstV has been isolated from diarrheic calves [1], initial attempts to experimentally reproduce the disease have been unsuccessful [2], and, in addition, neonatal calf diarrhea generally has a multifactorial origin; the aim of our study was not to establish an association between the presence of the virus and disease, but to determine the prevalence and genetic diversity of BoAstV circulating in dairy herds from Uruguay. The present survey showed a high prevalence of infection with a broad diversity of BoAstV. However, because there is great genetic diversity within the MAstV genus, and this may have implications in pathogenicity [17], further studies are needed to assess whether particular species in this genus are associated with enteric disease in cattle, as seems to be the case for bovine encephalitis linked to MAstV-13 [3,4]. To date, most BoAstVs are not assigned to any of the 19 MAstV species confirmed by the ICTV [9]. Nevertheless, recent studies indicate that BoAstV can be tentatively assigned to the species MAstV-13, MAstV-24, MAstV-28, MAstV-29, MAstV-30 and MAstV-33 [10].

The 432-nucleotide fragment of the polymerase gene of AstV [13] has been widely used for the screening of BoAstV throughout the world, both in fecal and central nervous system samples [11,18,19,20]; the RT-PCR has become a common tool for the detection of astrovirus because of its higher sensitivity compared with other techniques [21].

The phylogenetic diversity of Uruguayan BoAstV strains analyzed using the partial polymerase region was high, with the detection of 3/25 (12%) MAstV-28, 3/25 (12%) MAstV-33, and 19/25 (76%) of an unclassified MAstV species. BoAstV strains detected in Uruguay were closely related to BoAstVs detected in Brazil [18]. Several studies have classified BoAstVs in lineages based on the polymerase and/or capsid partial sequences [18,19,22,23,24]; most of the strains detected in these works clustered with the unclassified Uruguayan strains, indicating that this species is widely distributed worldwide (being probably the most prevalent) and demonstrating the importance of its correct classification.

Complete amino acid capsid sequence analyses are mandatory to establish new MAstV species [9]. In order to attempt to classify the divergent Uruguayan strains, capsid sequences were obtained. Unfortunately, probably due to the high variability in this genomic region, six partial sequences and only two complete capsid sequences were obtained. The phylogenetic analyses with the complete capsid sequences of the Uruguayan strains LVMS681 and LVMS2704 confirmed the results obtained with the polymerase region. Both strains clustered in a monophyletic group with other unclassified strains, both at the amino acid and the nucleotide level. These results were confirmed by the analysis of genetic differences in the complete capsid amino acid sequence, suggesting that the unclassified MAstV species found in this study should be assigned to a new MAstV species, as proposed by the ICTV.

The tentatively assigned species MAstV-13, MAstV-24, MAstV-29, MAstV-30, MAstV-33 and the unclassified MAstV species have p-distances within groups lower than 0.35, and between groups higher than this cut-off value determined for the standardized classification criteria [9] (Table 2). Surprisingly, MAstV-28 showed controversial results within this species, with p-distances at the amino acid level among strains higher than 0.350 (except between LC047790 and LC047798), the cut-off value for the species classification criteria. It is documented that, at initial stages of a viral emergence, sequences are separated almost exclusively by transient polymorphisms and not by fixed differences [25], and mutations accumulate more rapidly [26,27]. Probably, this higher genetic variation is due to incomplete purifying selection and some slightly deleterious mutations fall on external branches of phylogeny [26,27]. Some strains belonging to the MAstV-28 species were detected recently in nasopharyngeal samples [5], suggesting that these strains are adapting to tissues beyond the gastrointestinal tract [28]. Additional information of the strain LVMS1376 (from which the capsid region was partially amplified and classified as MAstV-28) was detected in a 3-month-old Holstein calf, which presumably died of pneumonia, although this data should be considered with caution, since the BoAstV detection and characterization was conducted from the intestinal content of this animal. The phylogenetic analysis revealed that MAstV-28 probably has a common ancestor with buffalo astrovirus, as previously described for other MAstV species [11], as well as ovine–bovine or porcine–bovine interspecies transmission events [23]. However, further studies are needed to better understand the molecular epidemiology of MAstVs.

The strain LVMS2692 showed controversial results; it clustered with the MAstV-unclassified strains in the polymerase genomic region used for screening, and with the MAstV-29 strain in the partial capsid region, suggesting coinfection and/or recombination. The region comprised between these two regions was amplified with primers BoAstV-F and BoAstV-CAP-U-33R1, but, unfortunately, chromatograms of both DNA strands showed mixed populations and could not be read, probably due to coinfection with the unclassified MAstV and MAstV-29. Coinfection has been described in BoAstV [13], and could be underestimated.

As the region of basic amino acids at the N-terminus of the capsid protein is thought to interact with the genomic RNA inside the virion [29], additional studies should be done to determine the implications of the insertion observed in the Uruguayan strains belonging to the unclassified MAstV.

Evidence indicates that the BoAstVs that belong to MAstV-13 are associated with neurological diseases and encephalitis [3,4,20], while the other species are not clearly associated to disease, but are excreted in feces [11,13,18,22,23], and/or detected in nasopharyngeal exudates [5]. On the other hand, it is important to study the consequence of asymptomatic cases in the epidemiology and transmission of the virus [17]. Interestingly, most of the strains from throughout the world [17,18,20,21,22] that could belong to the MAstV characterized in this work were mainly detected in diarrheic samples, although the role of BoAstV in the development of diarrhea in cattle needs further clarification.

## 5. Conclusions

BoAstV was highly prevalent in dairy calves in Uruguay; there was a high genetic diversity between strains and a MAstV species was characterized and classified. In addition, our analyses suggest that this species is the most prevalent worldwide, reinforcing the need for it to be correctly classified. Further studies are necessary to elucidate the possible enteropathogenicity of BoAstV species in cattle.

## Figures and Tables

**Table 1:** Primers used for complete capsid amplification.

Primer Name	5′–3′ Sequence	Genomic Region	Reference
BoAstV-F	GAYTGGACBCGHTWTGATGG	RdRp	[13]
BoAstV-R	KYTTRACCCACATNCCAA		
BoAstV-CAP-U-33F1	GCCCTCTATGGGAAACTCCT	Capsid	This study
BoAstV-CAP-U-33R1	GTMACCAKCCAKATWATYTC		
BoAstV-CAP-UF2	CAACARCCWGGGTTYATGAA	Capsid	This study
BoAstV-CAP-UR2	ATCCTCATCAGAGAGATCA		
BoAstV-CAP-UF3	TTGGAGATGGCRGAYGATGA	Capsid	This study
BoAstV-CAP-UR3	GCCAAATTAAATTAACTGG		
BoAstV-CAP-28F1	AGCCTCTGTGGGAAACTAGA	Capsid	This study
BoAstV-CAP-28R1	CCACCAVCCRCCCHTRAAAAGCCA		

**Table 2:** Estimates of evolutionary divergence between sequences at the amino acid level. The number of amino acid differences per site between sequences are shown (p-distance), on the left side the reference is the Uruguayan strain LVMS2704 (MN200263), and on the right side the reference is the Uruguayan strain LVMS681 (MN200262). Complete capsid sequences were used. Sequence names are: Accession number Host Strain name MAstV-species.

Uruguayan Strain LVMS2704 (MN200263)		Uruguayan Strain LVMS681 (MN200262)	
MAstV-Species	Distance	MAstV-Species	Distance
MN200262 BoAstV LVMS681 MAstV-X	0.01	MN200263 BoAstV LVMS2704 MAstV-X	0.01
LC047792 BoAstV Hokkaido12-18 MAstV-X	0.09	LC047792 BoAstV Hokkaido12-18 MAstV-X	0.09
LC047794 BoAstV Hokkaido12-27 MAstV-X	0.29	LC047794 BoAstV Hokkaido12-27 MAstV-X	0.29
LC047795 BoAstV Kagoshima1-2 MAstV-X	0.29	LC047795 BoAstV Kagoshima1-2 MAstV-X	0.29
LC047801 BoAstV Kagoshima2-52 MAstV-X	0.29	LC047801 BoAstV Kagoshima2-52 MAstV-X	0.29
LC047789 BoAstV Hokkaido11-7 MAstV-X	0.29	LC047789 BoAstV Hokkaido11-7 MAstV-X	0.29
LC047799 BoAstV Kagoshima2-24 MAstV-X	0.30	LC047799 BoAstV Kagoshima2-24 MAstV-X	0.30
LC047788 BoAstV Ishikawa9728 MAstV-X	0.31	LC047788 BoAstV Ishikawa9728 MAstV-X	0.31
HQ916314 BoAstV B170 MAstV-30	0.48	HQ916314 BoAstV B170 MAstV-30	0.48
HQ916316 BoAstV B76 MAstV-29	0.49	HQ916316 BoAstV B76 MAstV-29	0.49
HM756260 PoAstV 14-4 MAstV-31	0.52	HM756260 PoAstV 14-4 MAstV-31	0.52
KJ476833 BoAstV BAstGX-G1 MAstV-33	0.54	KJ476833 BoAstV BAstGX-G1 MAstV-33	0.54
KX645667 BaAstV LAP11-A0091 MAstV-X	0.54	KX645667 BaAstV LAP11-A0091 MAstV-X	0.54
LC047796 BoAstV Kagoshima1-7 MAstV-33	0.55	LC047796 BoAstV Kagoshima1-7 MAstV-33	0.54
HQ916313 BoAstV B18 MAstV-33	0.55	HQ916313 BoAstV B18 MAstV-33	0.55
KJ495986 PoAstV ExpPig-36 MAstV-32	0.55	HQ916317 BoAstV B76-2 MAstV-33	0.55
HQ916317 BoAstV B76-2 MAstV-33	0.55	KJ495986 PoAstV ExpPig-36 MAstV-32	0.55
KJ620979 BoAstV BAstV-GX7 MAstV-33	0.55	KJ620979 BoAstV BAstV-GX7 MAstV-33	0.55
KJ476834 BoAstV BAstGX-J7 MAstV-33	0.55	KJ476834 BoAstV BAstGX-J7 MAstV-33	0.55
KJ476832 BoAstV BAstGX-J27 MAstV-33	0.55	KJ476832 BoAstV BAstGX-J27 MAstV-33	0.55
KJ476836 BoAstV BAstGX-J8 MAstV-33	0.55	KJ476836 BoAstV BAstGX-J8 MAstV-33	0.55
KJ620980 BoAstV BAstV-GX27 MAstV-33	0.55	KJ620980 BoAstV BAstV-GX27 MAstV-33	0.55
KJ476835 BoAstV BAstGX-J22 MAstV-33	0.55	KJ476835 BoAstV BAstGX-J22 MAstV-33	0.55
KR868724 DrAstV DcAstV-274 MAstV-X	0.57	KR868724 DrAstV DcAstV-274 MAstV-X	0.56
HQ916315 BoAstV B34 MAstV-28	0.60	HQ916315 BoAstV B34 MAstV-28	0.60
LC047787 BoAstV Ishikawa24-6 MAstV-28	0.61	LC047787 BoAstV Ishikawa24-6 MAstV-28	0.61
LC047797 BoAstV Kagoshima2-3-1 MAstV-28	0.61	LC047797 BoAstV Kagoshima2-3-1 MAstV-28	0.61
LC047800 BoAstV Kagoshima2-38 MAstV-28	0.65	LC047800 BoAstV Kagoshima2-38 MAstV-28	0.65
KT963071 BoAstV 715 MAstV-28	0.66	KT963071 BoAstV 715 MAstV-28	0.66
LC047798 BoAstV Kagoshima2-3-2 MAstV-28	0.67	LC047798 BoAstV Kagoshima2-3-2 MAstV-28	0.67
KP264970 BoAstV BSRI-1 MAstV-28	0.67	KP264970 BoAstV BSRI-1 MAstV-28	0.67
LC047790 BoAstV Hokkaido11-55 MAstV-28	0.68	LC047790 BoAstV Hokkaido11-55 MAstV-28	0.68
JQ408745 MuAstV TF18LM MAstV-X	0.70	JX684071 PoAstV US-P2011-1 MAstV-26	0.70
JX544746 MuAstV STL 4 MAstV-X	0.70	JQ408745 MuAstV TF18LM MAstV-X	0.70
JX684071 PoAstV US-P2011-1 MAstV-26	0.70	JX544746 MuAstV STL 4 MAstV-X	0.70
KU764486 PoAstV 15-12 MAstV-26	0.71	KU764486 PoAstV 15-12 MAstV-26	0.71
JX556692 PoAstV IL135 MAstV-27	0.72	JX556692 PoAstV IL135 MAstV-27	0.72
KM017742 CaAstV FAstV-D2 MAstV-2	0.74	KM017742 CaAstV FAstV-D2 MAstV-2	0.74
L23513 HuAstV Oxford-1 MAstV-1	0.74	JN592482 OvAstV OAstV-2 MAstV-24	0.74
JN592482 OvAstV OAstV-2 MAstV-24	0.74	LC201619 PoAstV Ishi-Im1-1 MAstV-24	0.74
LC201619 PoAstV Ishi-Im1-1 MAstV-24	0.74	L23513 HuAstV Oxford-1 MAstV-1	0.74
HM450381 RatAstV RS118 MAstV-25	0.75	HM450381 RatAstV RS118 MAstV-25	0.75
L06802 HuAstV Oxford-2 MAstV-1	0.75	LC047793 BoAstV Hokkaido12-25 MAstV-24	0.75
LC047793 BoAstV Hokkaido12-25 MAstV-24	0.76	GQ914773 PoAstV Shanghai MAstV-3	0.75
GQ914773 PoAstV Shanghai MAstV-3	0.76	L06802 HuAstV Oxford-2 MAstV-1	0.76
JN420351 CslAstV 4 1136 MAstV-11	0.76	KR349491 DoAstV Grav MAstV-5	0.76
KR349491 DoAstV Grav MAstV-5	0.76	JN420351 CslAstV 4 1136 MAstV-11	0.76
JN420354 CslAstV 1169 MAstV-4	0.76	JN420354 CslAstV 1169 MAstV-4	0.76
FJ222451 HuAstV MLB1 MAstV-6	0.77	FJ222451 HuAstV MLB1 MAstV-6	0.77
FJ571068 BaAstV LS11 MAstV-17	0.78	JF729316 RabAstV 2208 MAstV-23	0.78
JF729316 RabAstV 2208 MAstV-23	0.78	FJ571068 BaAstV LS11 MAstV-17	0.78
EU847144 BaAstV AFCD57 MAstV-14	0.78	EU847144 BaAstV AFCD57 MAstV-14	0.78
FJ571066 BaAstV LD77 MAstV-15	0.78	FJ571066 BaAstV LD77 MAstV-15	0.78
FJ571071 BaAstV DX19 MAstV-19	0.79	FJ571071 BaAstV DX19 MAstV-19	0.79
FJ973620 HuAstV VA1 MAstV-9	0.79	FJ973620 HuAstV VA1 MAstV-9	0.79
EU847145 BaAstV AFCD11 MAstV-16	0.79	GQ502193 HuAstV VA2 WD0680 MAstV-8	0.79
GQ502193 HuAstV VA2 WD0680 MAstV-8	0.79	EU847145 BaAstV AFCD11 MAstV-16	0.79
KF233994 BoAstV NeuroS1 MAstV-13	0.79	KM035759 BoAstV CH13 MAstV-13	0.79
KM035759 BoAstV CH13 MAstV-13	0.79	KF233994 BoAstV NeuroS1 MAstV-13	0.79
AY179509 MiAstV MAstV-10	0.80	AY179509 MiAstV MAstV-10	0.80
FJ890355 BdAstV Bd1 MAstV-7	0.80	JF755422 MoAstV M-52 MAstV-20	0.80
JF755422 MoAstV M-52 MAstV-20	0.80	FJ890355 BdAstV Bd1 MAstV-7	0.80
FJ571067 BaAstV LD71 MAstV-12	0.80	FJ571067 BaAstV LD71 MAstV-12	0.80
Y15937 OvAstV Snodgrass MAstV-13	0.80	Y15937 OvAstV Snodgrass MAstV-13	0.80
GU985458 MiAstV SMS MAstV-21	0.80	GU985458 MiAstV SMS MAstV-21	0.80
KT956903 BoAstV CH15 MAstV-13	0.80	EU847155 BaAstV AFCD337 MAstV-18	0.80
LN879482 BoAstV BH89/14 MAstV-13	0.80	KT956903 BoAstV CH15 MAstV-13	0.80
EU847155 BaAstV AFCD337 MAstV-18	0.81	LN879482 BoAstV BH89/14 MAstV-13	0.80

## Supplementary Materials

The following are available online at https://www.mdpi.com/1999-4915/12/1/32/s1. Figure S1: Maximum-likelihood tree constructed with complete capsid nucleotide sequences of MAstVs, Figure S2: Maximum-likelihood tree constructed with partial capsid amino acid sequences, Table S1: Partial polymerase sequences used for the phylogenetic analysis, and Table S2: Complete capsid sequences used for the MAstV species classification.
