# Supplementary figures and images for: Bovine Astrovirus Surveillance in Uruguay Reveals High Detection Rate of a Novel Mamastrovirus Species
Source: Viruses. 2019 Dec 27;12(1):32. doi: 10.3390/v12010032 (PMC7019600; doi:10.3390/v12010032)

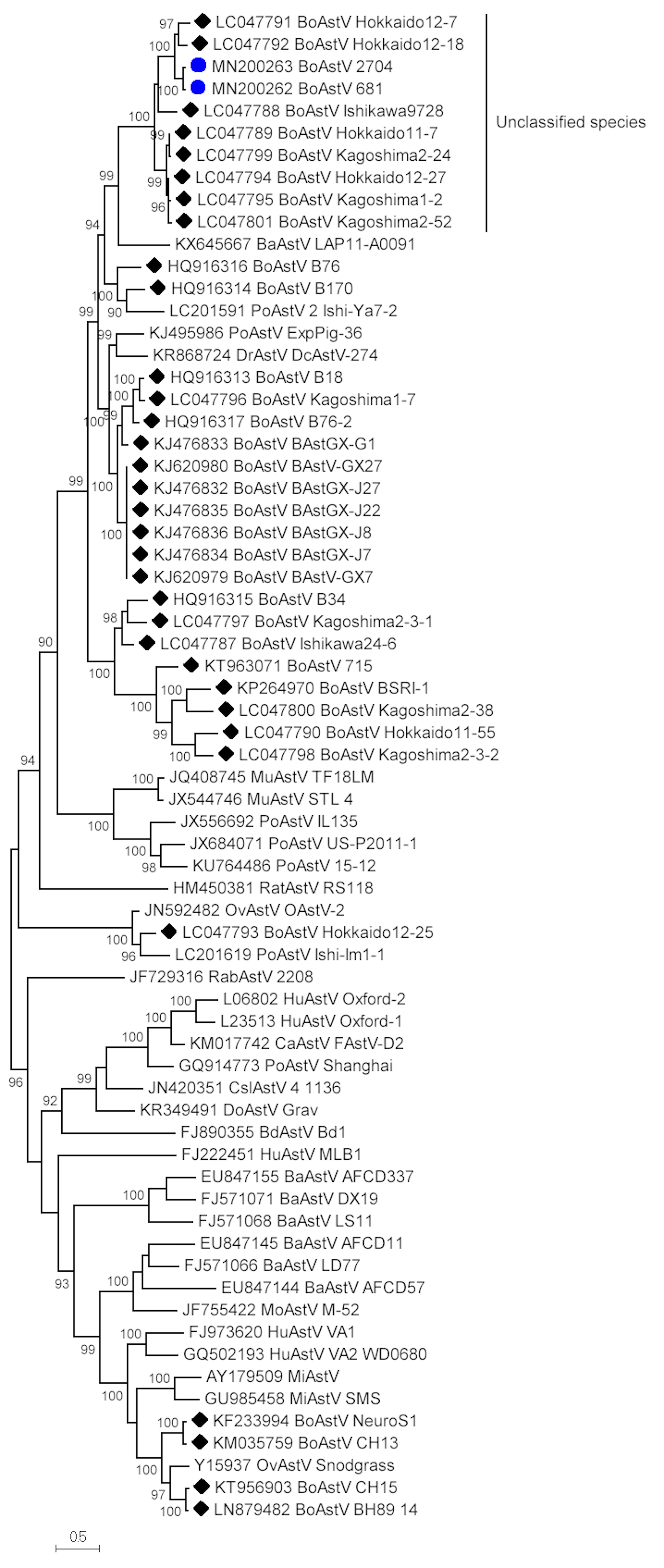

Supplement: Supplementary file 1 [file viruses-12-00032-s001.zip › Supplemental Material/Figure S1.tif]

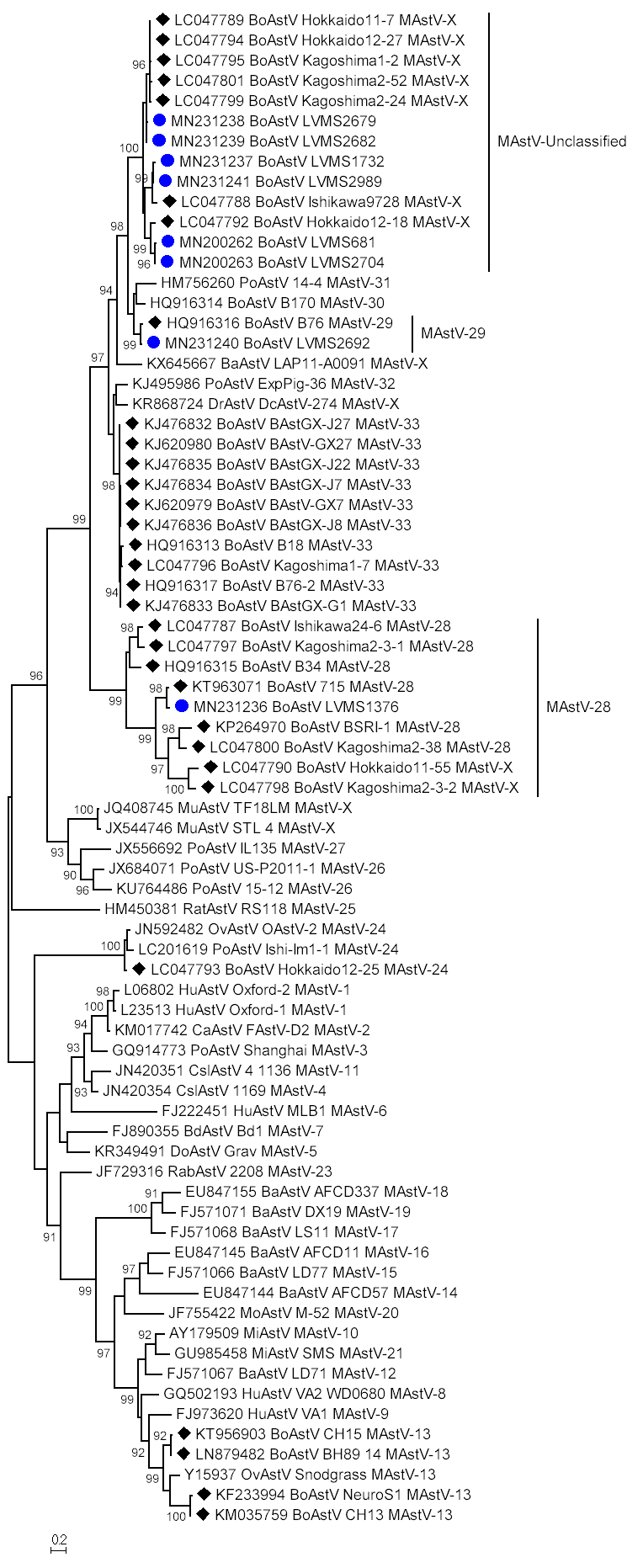

Supplement: Supplementary file 1 [file viruses-12-00032-s001.zip › Supplemental Material/Figure S2.tif]
